# Roles and Preliminary Mechanism of Tobacco *cis*-Abienol in Inducing Tomato Resistance against Bacterial Wilt

**DOI:** 10.3390/ijms241512226

**Published:** 2023-07-31

**Authors:** Yuqing Sun, Zuqing Gui, Ning Yan, Qian Wang, Zhongfeng Zhang, Hongbo Zhang, Feifei Sun, Xiao Han, Yongmei Du

**Affiliations:** 1 Tobacco Research Institute of Chinese Academy of Agricultural Sciences, Qingdao 266101, China; 15211260220@163.com (Y.S.); gzgzgzq1201@163.com (Z.G.);; 2 Graduate School of Chinese Academy of Agricultural Sciences, Beijing 100081, China

**Keywords:** *cis*-abienol, bacterial wilt, induced resistance, functional mechanism

## Abstract

Bacterial wilt negatively impacts the yield and quality of tomatoes. *cis*-Abienol, a labdane diterpenoid abundantly produced in the trichome secretion of *Nicotiana* spp., can induce bacterial wilt resistance in plants; however, study on its practical application and acting mechanism is very limited. This study established the application conditions of *cis*-abienol for inducing tomato bacterial wilt resistance by pot-inoculation experiments and investigated the underlying mechanism by determining the physio-biochemical indexes and transcriptomic changes. The results showed that applying *cis*-abienol to the roots was the most effective approach for inducing tomato bacterial wilt resistance. The optimal concentration was 60 μg/mL, and 2–3 consecutive applications with 3–6 days intervals were sufficient to induce the bacterial wilt resistance of tomato plants. *cis*-Abienol could enhance the antioxidant enzyme activity and stimulate the defensive signal transduction in tomato roots, leading to the upregulation of genes involved in the mitogen-activated protein kinase cascade. It also upregulated the expression of *JAZ* genes and increased the content of jasmonic acid (JA) and salicylic acid (SA), which control the expression of flavonoid biosynthetic genes and the content of phytoalexins in tomato roots. *cis*-Abienol-induced resistance mainly depends on the JA signalling pathway, and the SA signalling pathway is also involved in this process. This study established the feasibility of applying the plant-derived terpenoid *cis*-abienol to induce plant bacterial wilt resistance, which is of great value for developing eco-friendly bactericides.

## 1. Introduction

Bacterial wilt of crops is a soil-borne disease caused by *Ralstonia solanacearum* and triggers serious crop diseases worldwide [1]. Tomato (*Solanum lycopersicum*), one of the most important vegetable crops with an annual output of approximately 160 million tons, is always threatened by bacterial wilt, which often leads to large-scale wilt and death of tomato plants and significant economic losses of tomato production [2]. Currently, no tomato cultivar has been identified to have effective resistance to bacterial wilt. Meanwhile, the overuse of chemicals causes serious environmental pollution, and recently developed microbial control methods are not stable enough due to the diversity and adaptive abilities of microorganisms [3,4]. Therefore, more effective methods are required to reduce bacterial wilt disease during tomato cultivation practice. Although the losses of crop yield caused by bacterial wilt can be reduced to a certain extent by comprehensive measures, such as chemical application, microbial control, planting disease-resistant varieties, adjusting soil microorganisms, regulating the period of crop transplanting, and rational rotation [5,6], the occurrence of bacterial wilt still cannot be effectively controlled.

Plant-resistance-inducing technology is a new way to control crop diseases because of its advantages of security, broad spectrum, and persistence. Immune elicitors can enhance plant resistance by stimulating oxidative metabolism related to disease resistance and increasing the accumulation of secondary metabolites [7]. The application of thymol and acibenzolar-s-methyl in the field could induce an increase in polyphenol oxidase (PPO) and beta-glucosidase activities in tomato plants to reduce the occurrence of bacterial wilt [8,9]. Silicon application in tomatoes can increase the activities of peroxidase (POD) and phenylalanine ammonia-lyase (PAL) and the contents of salicylic acid (SA) and jasmonic acid (JA), leading to lignin deposition that can alleviate the harm of bacterial wilt [10]. Treatment with seaweed polysaccharides increases the content of JA in the xylem of tomato and enhances the resistance against bacterial wilt, which may be related to the damage-associated molecular pattern reaction pathway [11]. L-arabinose could reduce the damage of bacterial wilt on tomatoes by upregulating the defence genes related to SA and ethylene responses, such as *PR-1a/GLUa/OLP*, in tomato plants [12].

Tobacco (*Nicotiana tabacum* L.) is an industrial crop grown worldwide. Recently, the demand for tobacco as a raw material for cigarettes significantly decreased due to the implementation of international tobacco control agreements. This has presented great challenges for tobacco cultivation and promoted studies on the alternative usage of tobacco [13]. The surface of tobacco aerial tissues is densely covered with glandular trichomes, and the glandular trichome secretions are secondary metabolites with unique molecular structures and defensive bioactivities for developing botanical pesticides [14]. *cis*-Abienol is the most abundant labdane diterpenoid in tobacco trichome secretions and is mainly found in oriental tobacco, sun-cured tobacco, and cigar tobacco [15]. Previous studies have shown that *cis*-abienol can inhibit the occurrence of bacterial wilt in crops by root treatment [16]. Fujimoto et al. (2015) [17] demonstrated that sclareol, which has a similar chemical structure to *cis*-abienol, can upregulate the *Arabidopsis thaliana* ATP-binding cassette transporter gene *AtPDR12* and improve the resistance to bacterial wilt. Therefore, we speculated that *cis*-abienol and sclareol may have similar bioactivity. However, suitable application conditions (such as concentration, time interval, and times) of *cis*-abienol have not been established to date, and the mechanism underlying *cis*-abienol-induced tomato resistance to bacterial wilt remains to be uncovered. This study aimed to investigate the suitable conditions for inducing tomato resistance against bacterial wilt using *cis*-abienol to explore the preliminary mechanism underlying this process. Our findings provide a basis for the application of *cis*-abienol as an immune elicitor of *R. solanacearum* resistance in tomato plants.

## 2. Results

### 2.1. In Vitro Activity of cis-Abienol against R. solanacearum

**Figure 1:**
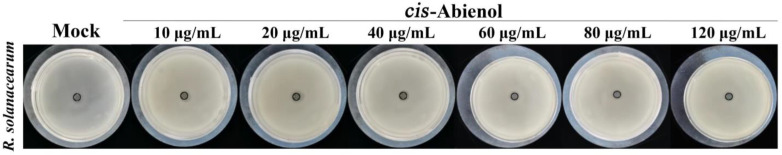
Effect of different concentrations of *cis*-abienol on the growth of *R. solanacearum* in vitro.

We conducted an experiment to determine if *cis*-abienol has a direct inhibitory effect on *R. solanacearum* in vitro. As shown in Figure 1, we found that at the concentrations of 10–120 μg/mL, *cis*-abienol did not significantly inhibit the growth of *R. solanacearum*. This result is consistent with the findings by Seo and colleagues (2012) [16].

### 2.2. Optimal Conditions for Tomato Seedlings to Acquire a cis-Abienol-Induced Resistance to Bacterial Wilt

#### 2.2.1. Determination of Optimal Organs for Resistance Induction

**Figure 2:**
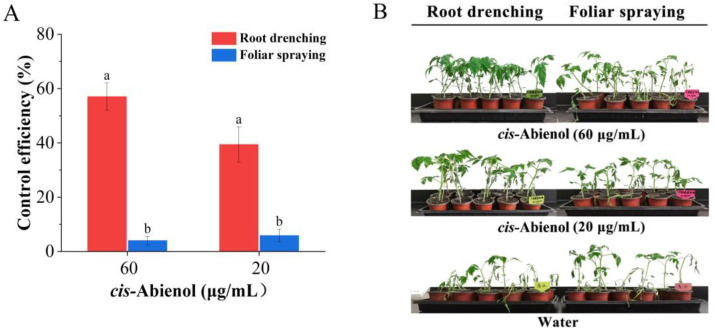
Response of tomato to bacterial wilt after *cis*-abienol treatment on different organs. (**A**) The effects of *cis*-abienol treatment on tomato bacterial wilt resistance. Values are represented as a percentage of plants that acquired resistance after treatment. The method used to calculate the control efficiency is described in Section 4.4.1. Bars indicate mean ± standard deviation (SD) (*n* = 4). Different lowercase letters denote statistical significance determined via Duncan’s multiple range test (*p* < 0.05). (**B**) Representative images of tomato plants after the *cis*-abienol treatment.

As shown in Figure 2, the control efficiency of root drenching with *cis*-abienol on tomato bacterial wilt is significantly higher than that observed in the plants treated by foliar spraying of *cis*-abienol (*p* < 0.01), and no significant difference (*p* > 0.05) is observed between the foliar spraying of *cis*-abienol and foliar spraying of water on tomato bacterial wilt. Therefore, the suitable organ for *cis*-abienol application for inducing tomato bacterial wilt resistance is the root.

#### 2.2.2. Determination of Optimal *cis*-Abienol Concentrations for Resistance Induction

**Figure 3:**
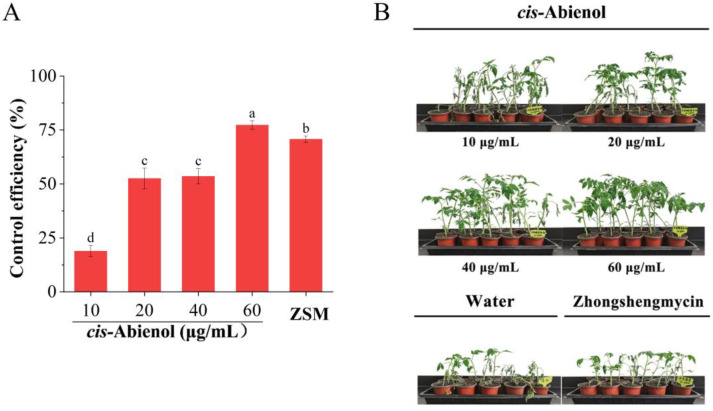
Induction of tomato bacterial wilt resistance by *cis*-abienol. (**A**) Induction of tomato bacterial wilt resistance with different concentrations of *cis*-abienol. ZSM represents positive control zhongshengmycin (50 μg/mL). The effectiveness of *cis*-abienol is represented as the percentage of tomato plants that acquired resistance after *cis*-abienol treatment Bars indicate mean ± SD (*n* = 4). Different lowercase letters denote statistical significance as determined via Duncan’s multiple range test (*p* < 0.05). (**B**) Representative images of tomato seedlings after the *cis*-abienol treatment.

As shown in Figure 3, the bacterial wilt resistance of tomato plants increases after *cis*-abienol treatment, and the induced resistance is accentuated to higher levels as the *cis*-abienol concentrations increase. Notably, once the concentration of *cis*-abienol reached 60 μg/mL, the induced bacterial wilt resistance was significantly higher than that in the treatment with a lower concentration of *cis*-abienol (*p* < 0.05), and it was even higher than the positive control treatment with ZSM (zhongshengmycin; *p* < 0.05). The optimal concentrations of immune elicitors benzothiadiazole (BTH) and 2,6-dichloroisonicotinic acid [18] for inducing bacterial wilt resistance in crops was reported as 200 μg/mL, that for riboflavin [19] was reported as 400 μg/mL, and that for SA [20] was 500 μg/mL. In comparison, the concentration of *cis*-abienol for inducing bacterial wilt resistance is much lower, indicating higher eliciting activity.

#### 2.2.3. Determination of Optimal Intervals of *cis*-Abienol Application for Resistance Induction

**Figure 4:**
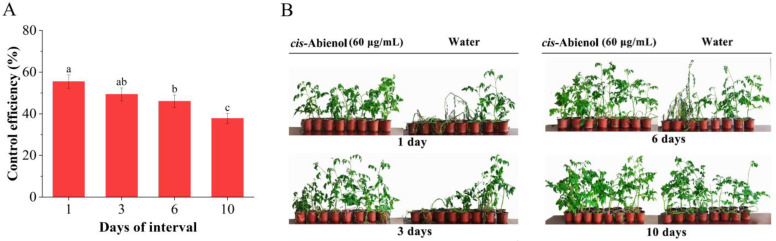
Effect of different intervals of *cis*-abienol (60 μg/mL) application on the resistance of tomato seedlings to bacterial wilt. The control efficiency is represented as the percentage of tomato seedlings that acquired resistance after *cis*-abienol treatment. (**A**) Bars indicate the mean ± SD (*n* = 4). Different lowercase letters denote statistical significance as determined via Duncan’s multiple range test (*p* < 0.05). (**B**) Representative images of tomato seedlings after the *cis*-abienol treatment.

As shown in Figure 4, the control efficiency of *cis*-abienol on tomato bacterial wilt resistance decreases with time extending after inoculation. The control efficiency of *R. solanacearum* inoculated with 3 days of interval is not significantly different from that inoculated with 1 or 6 days of intervals (*p* > 0.05). However, the effectiveness of treatment with 1–6 days of intervals is significantly higher than that of treatments with 10 days of intervals (*p* < 0.05). Therefore, to maximise tomato yields and their associated economic benefits, *cis*-abienol should be applied every 3–6 days. When the resistance genes are expressed, and the defence products are accumulated after the immune elicitor treatment, the plant shows resistance to the pathogen. However, the duration of the resistance gene expression varies with the plants and immune elicitors. For example, the optimal interval for silicon-induced resistance to bacterial wilt in tomatoes is 3 days [21], and the ideal interval for scopolamine-induced resistance expression in tobacco is also 3 days [22,23].

In this study, the effect of *cis*-abienol on tomato seedlings’ resistance to bacterial wilt significantly decreased 10 days after treatment (*p* < 0.05), which may be due to the decrease in the induction information of tomato seedlings after being stimulated. After the 10th day of treatment with *cis*-abienol, the accumulation of defence reaction products is reduced, leading to a significant reduction in resistance to bacterial wilt (*p* < 0.05).

#### 2.2.4. Determination of Suitable Number of *cis*-Abienol Applications for Resistance Induction

**Figure 5:**
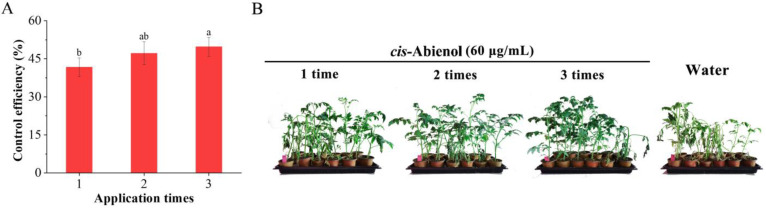
Effect of different numbers of *cis*-abienol (60 μg/mL) treatments on tomato resistance to bacterial wilt. The relative control efficiency is represented as the percentage of tomato seedlings that acquired resistance. (**A**) Bars indicate the mean ± SD (*n* = 4). Different lowercase letters denote statistical significance as determined via Duncan’s multiple range test (*p* < 0.05). (**B**) Representative images of tomato seedlings after the *cis*-abienol treatment.

As shown in Figure 5, the control efficiency of *cis*-abienol on bacterial wilt of tomato seedlings showed an upward trend with the increase in induction times, and no significant difference is observed between two and three treatments (*p* > 0.05), but the relative control efficiency is significantly higher (*p* < 0.05) than that after one treatment. Therefore, the suitable number of *cis*-abienol treatments is 2–3. After several treatments, the duration of plant resistance is prolonged. However, the increases in induced resistance are limited at a certain point. For example, the optimal application of scopolamine to tobacco bacterial wilt was 2–3 times, and the optimal application of 5% aminooligosaccharides to cotton fusarium wilt was 2–3 times [24], which is similar to the results of this study.

### 2.3. Effect of cis-Abienol Treatment on the Growth and Development of Tomato Seedlings

**Figure 6:**
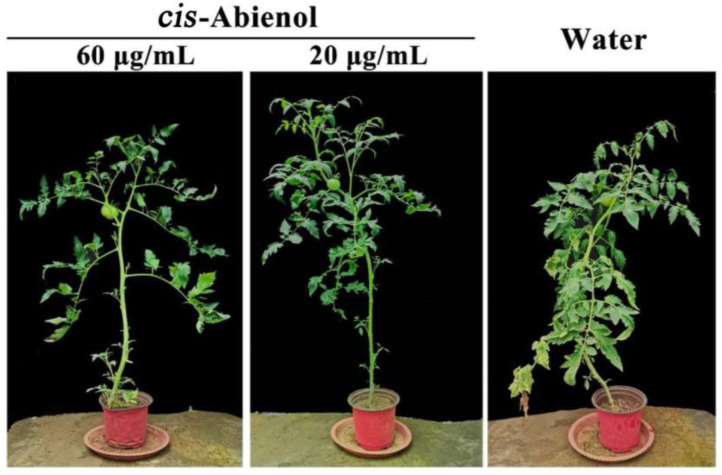
Representative images of tomato seedlings after the *cis*-abienol treatment.

Table 1 shows that the time from seed sowing to the flowering period of tomato plants after *cis*-abienol induction was the same as that observed in the control treatment with water. Figure 6 shows that representative images of tomato seedlings after the *cis*-abienol treatment. Although the fresh weight of the above-ground and underground parts of tomato plants is slightly higher after *cis*-abienol treatment than after the control treatment with water, the difference is not significant (*p* > 0.05). Therefore, *cis*-abienol treatment has no significant effect on the growth and development of tomato plants.

### 2.4. Effect of cis-Abienol Treatment on the Activities of Defence Enzymes, Phytoalexin, and Phytohormone Content in Tomato Roots

#### 2.4.1. Effects on the Activities of Defence Enzymes

**Figure 7:**
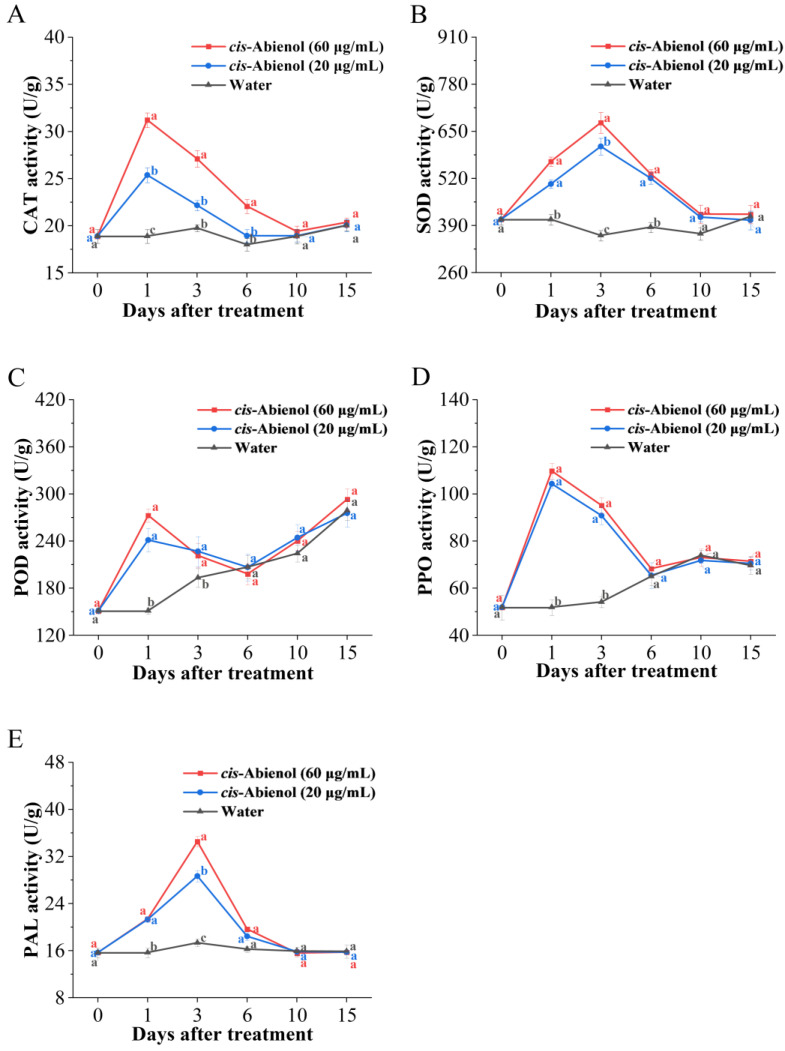
Effects of *cis*-abienol on the activities of CAT (**A**), SOD (**B**), POD (**C**), PPO (**D**), and PAL (**E**) in tomato roots. Bars indicate the mean ± SD (*n* = 4. Different lowercase letters denote statistical significance as determined via Duncan’s multiple range test (*p* < 0.05).

As shown in Figure 7, the activities of defence enzymes in tomato roots increase after *cis*-abienol treatment and subsequently decrease 15 days later, and a significant correlation with *cis*-abienol concentration is observed. The activities of catalase (CAT) and superoxide dismutase (SOD) after *cis*-abienol treatment are significantly higher (*p* < 0.05) than those after 1–6 days of the control treatment (Figure 7A,B). Moreover, the activities of POD, PPO, and PAL after *cis*-abienol treatment are significantly higher (*p* < 0.05) than those after 1–3 days of the control treatment (Figure 7C–E). After 10 days of *cis*-abienol treatment, the activities of defence enzymes in tomato roots decreases to the same level as in that of the control treatment.

The variation in defence enzyme activity in plants is closely related to plant pathogen resistance. CAT and SOD can maintain the reactive oxygen species (ROS) balance and repair the damage caused by oxygen free radicals. Meanwhile, PAL and POD are closely related to the synthesis of flavonoids and lignin, and PPO can oxidise flavonoids in plants into quinones that have antibacterial properties [25]. Thus, the findings of this study indicate that *cis*-abienol could promote ROS balance and increase the activities of defence enzymes related to phytoalexin biosynthesis in tomatoes.

#### 2.4.2. Effects on Phytoalexin Contents

**Figure 8:**
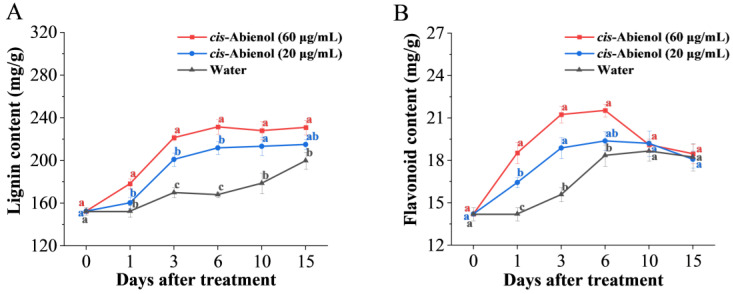
Effect of *cis*-abienol on the lignin (**A**) and flavonoid (**B**) content in tomato root. Bars indicate the mean ± SD (*n* = 4). Different lowercase letters denote statistical significance as determined via Duncan’s multiple range test (*p* < 0.05).

As shown in Figure 8, the lignin and flavonoid contents in tomato roots after *cis*-abienol treatment are significantly higher (*p* < 0.05) than those after 1 day of control treatment and show a significant correlation with *cis*-abienol concentration. The content of lignin and flavonoids increases rapidly after 1–3 days of *cis*-abienol treatment and reaches the highest level after 6 days of treatment. Subsequently, the lignin content continuously increases until 15 days later, whereas the flavonoid content slowly decreases until 10 days later.

As a product of the phenylpropane pathway, lignin can form a mechanical barrier, which weakens the diffusion and proliferation of pathogenic bacteria in plants. At the same time, it can be used as a precursor for lignin biosynthesis to increase plant lignification degrees and further enhance plant disease resistance. The above results show that the contents of lignin and flavonoids increase in tomato roots after treatment with *cis*-abienol. Tomato resistance to bacterial wilt is high when the total phytoalexin content in tomatoes remains at a high level for 3–6 days. The results are consistent with the above finding that the control efficiency (Figure 4) of bacterial wilt by *cis*-abienol treatment with 3–6 days of intervals.

#### 2.4.3. Effect of *cis*-Abienol Treatment on Plant Hormone Content

**Figure 9:**
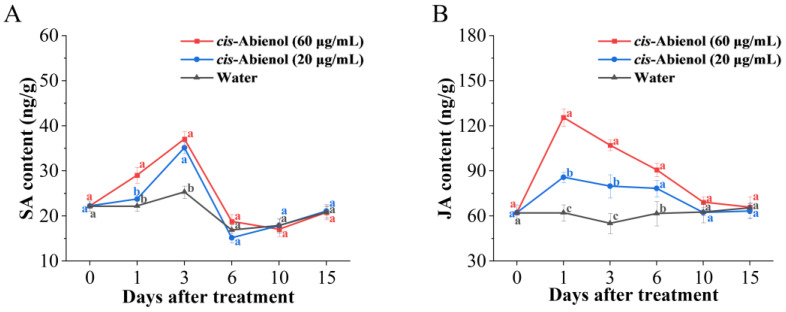
Effect of *cis*-abienol on SA (**A**) and JA (**B**) content in tomato root. Bars indicate the mean ± SD (*n* = 4). Different lowercase letters denote statistical significance as determined via Duncan’s multiple range test (*p* < 0.05).

As shown in Figure 9, the contents of SA and JA significantly increase after *cis*-abienol treatment and are significantly correlated with the concentration of *cis*-abienol. The content of SA in tomato roots is higher than that in control plants after 1–3 days of treatment. The SA content peaks after 3 days of treatment when it was 1.48 times higher than that in control plants. Meanwhile, the content of JA is higher than that in control plants after 1–6 days of *cis*-abienol treatment and reaches the highest level after 1 day of treatment, when it was 2.01 times that in control plants. After *cis*-abienol treatment, the extent and duration of JA content increase is greater than that of SA.

With regard to long-term adaptations, plants have evolved powerful signalling networks and multilevel defence mechanisms to deal with environmental threats. The SA- and JA signalling pathways regulate phytoalexin biosynthesis and play an important role in plant resistance to pathogen infection [26]. Differences in the interaction systems between immune elicitors and host plants may lead to differences in disease resistance and defence response pathways. In this study, the *cis*-abienol-induced increase in JA content in tomato roots was higher than that of SA, and the duration of JA increase was also longer than that of SA. This may have resulted from the resistance pattern induced by *cis*-abienol, which depends on JA-mediated induced systemic resistance (ISR) and is accompanied by SA-mediated systemic acquired resistance (SAR).

### 2.5. Analysis of Differentially Expressed Genes Induced by cis-Abienol in Tomato

#### 2.5.1. Statistics of Differentially Expressed Genes

**Figure 10:**
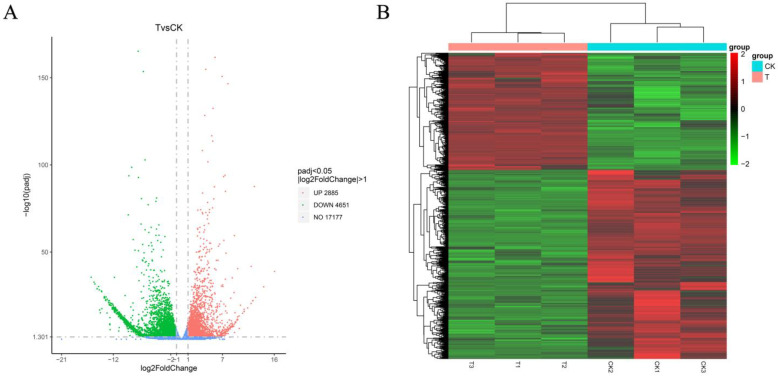
Volcano plot (**A**) showing gene expression levels determined using high-throughput RNA sequencing. Dotted lines represent the *p*-value and fold-change classification thresholds for differential expression. Red (“up”) and green (“down”) dots represent DEGs with increased and decreased expression, respectively. Genes indicated by blue dots (“no”) were not classified as differentially expressed genes. Heatmap (**B**) of all DEGs. Colour indicates the level of relative content of each DEG, from green (low) to red (high). CK indicates the control treatment with water, and T indicates the *cis*-abienol (60 μg/mL) treatment.

Through a comparative analysis of gene expression between the groups of *cis*-abienol treatment (T) and water treatment (CK), 7536 differentially expressed genes (DEGs) were obtained under the condition of DESeq2 (*p* < 0.05) |log_2_Fold Change| > 1, of which 2885 DEGs were upregulated, and 4651 DEGs were downregulated (Figure 10A, Table S1). According to the heatmap (Figure 10B), the total DEGs are significantly clustered (*p* < 0.05) before and after treatment with *cis*-abienol (Table S2). High reliability was determined by better internal repeatability of each treatment and higher segregation between treatments.

#### 2.5.2. Enrichment of Gene Ontology and Genes of Different Metabolic Pathways

**Figure 11:**
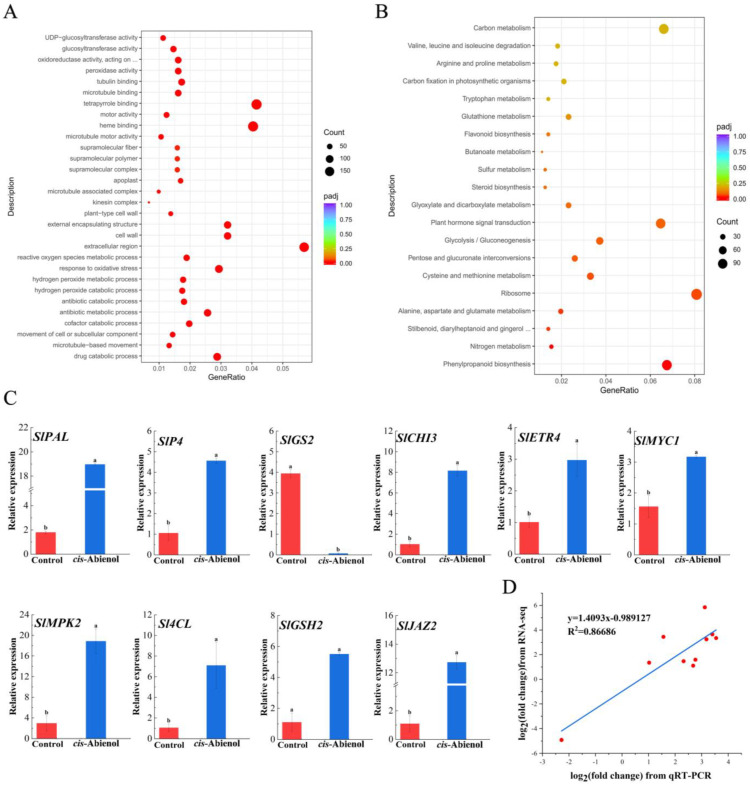
GO (**A**) and KEGG (**B**) enrichment analysis of DEGs and relative expression levels of selected DEGs determined by qRT-PCR (**C**). GeneRatio indicates the ratio of DEGs mapped to a certain pathway over the total number of genes mapped to the same pathway. Results from the GO pathway enrichment analysis for DEGs; the 30 most enriched pathways are listed (left). Results from the KEGG pathway enrichment analysis for DEGs; the 20 most-enriched pathways are listed. Bubble colour indicates the padj. Bubble size corresponds to the gene number enriched in the pathway. In (**C**), 10 defence-related DEGs were selected and determined using qRT-PCR. The bars indicate the mean ± SD (*n* = 3). Correlation using a linear model (**D**) between expression measurements obtained by qRT-PCR and RNA-seq. Different lowercase letters in subfigures (**C**,**D**) denote statistical significance as determined via Duncan’s multiple range test (*p* < 0.05).

As shown in Figure 11A (Table S3), the top 30 Gene Ontology (GO) entries with the highest enrichment were analysed for DEGs, including cell wall organisation or biogenesis, cell membrane composition and function, intracellular component transport, ATP synthesis, activities of various oxidoreductases and biosynthesis kinases, oxidative stress response, chemical homeostasis, and DNA replication and synthesis. All sequences were compared with the Genes and Genomes (KEGG) database to understand the metabolic pathways in which these DEGs are involved (Figure 11B, Table S4). The pathways of significant gene enrichment mainly involved flavonoid metabolism, phenylpropanoid metabolism, MAPK signalling cascade, glutathione metabolism, plant hormone signal transduction, and pyridine alkaloid metabolism. The results of GO and KEGG enrichment show that *cis*-abienol mainly affects the MAPK cascade, hormone signalling, and phenylpropane metabolism in tomato roots, which are related to plant disease resistance.

Details on the DEGs significantly enriched in the pathway related to disease resistance in tomato roots after *cis*-abienol application are provided in Table 2. ROS and Ca^2+^ signal transduction are important pathways for extracellular signal transduction to the cells. The calcium-binding protein gene, Solyc01g058720.3 (calcium-binding protein CP1, log_2_FC = 2.30), of *cis*-abienol-treated tomato roots was upregulated. Combined with the results of the upregulation of enzymes related to ROS metabolism, such as POD, CAT and SOD, these results show that *cis*-abienol can regulate ROS balance in tomato roots and induce signal transduction.

MAPK cascades are significant in induced plant resistance, as they transmit intracellular signals induced by changes in ROS content to the nucleus. Tomato roots control the upregulation of MAPK cascade-related DEGs, such as the important substrate protease gene *Sl*MPK2 (mitogen-activated protein kinase 2, log_2_FC = 1.11) and the signalling synthesis genes *Sl*MYC1 (transcription factor MYC1, log_2_FC = 1.35) and *Sl*CHI3 (acidic 26 kDa endochitinase precursor, log_2_FC = 3.25). These results indicate that *cis*-abienol could induce the enhancement of the MAPK cascade in tomato roots.

SA and JA signalling pathways are closely related to plant disease resistance. In the DEGs encoding plant hormone synthesis, JA synthesis-related genes *SlJAZ2* (jasmonate ZIM-domain protein 2, log_2_FC = 3.35), *SlJAZ3* (log2FC = 4.56) and ET synthesis genes *SlETR4* (ethylene receptor ETR4 precursor, log_2_FC = 3.45), *SlEREB4* (log_2_FC = 3.70) were upregulated, SA synthesis-related genes *SlP4* (pathogenesis-related leaf protein 4 precursor, log_2_FC = 5.85) was upregulated, while GA biosynthesis genes *SlGAI* (log_2_FC = −1.20) was downregulated. The results show that *cis*-abienol enhances SA and JA signal transduction in tomato roots and have a wide influence on the JA signalling pathway. Downregulation of the GA biosynthesis gene *SlGAI*, which may be caused by the interaction of GA and JA signalling pathways, can affect the energy dissipation of other growth pathways.

The phenylpropane metabolic pathway is an important pathway for phytoalexin synthesis in plants. In this pathway, encoding key enzyme gene *SlPAL* (Phenylalanine ammonia-lyase, log_2_FC = 3.35) for tomato lignin synthesis, flavonoid synthesis key enzyme genes *Sl4CL* (4-coumarate--CoA ligase, log_2_FC = 3.35) and *SlCHI1* (chalcone--flavonone isomerase, log_2_FC = 2.27) were upregulated, and the glutathione synthetase gene *SlGSH2* (glutathione synthetase, chloroplastic, log_2_FC = 1.47) was upregulated, indicating that *cis*-abienol promoted the biosynthesis of phytoalexins such as lignin and flavonoids in tomato roots.

In addition, other genes involved in disease resistance, such as the sterol 14 α-demethylase gene *SlCYP51* (sterol C14-demetylase, log_2_FC = 1.06), which is an important membrane protein, were identified; synthetic sesquiterpene and triterpenoid antitoxin gene *SlSQS1* (squalene synthase, log_2_FC = 1.63), *SlTPS33* (viridiflorene synthase, log_2_FC = 2.48), and *SlTTS1* (beta-amyrin synthase, log_2_FC = 4.45) were upregulated, indicating that *cis*-abienol affected the metabolic pathways related to disease resistance in tomato roots.

Ten defence-associated DEGs (*SlPAL*, *SlP4*, *SlGS2*, *SlCHI3*, *SlETR4*, *SlMYC1*, *SlMPK2*, *Sl4CL*, *SlCSH2*, and *SlJAZ2*) were selected for qRT-PCR analysis to validate the accuracy of RNA-seq data (Figure 11C). The transcription levels of the genes determined by qRT-PCR are consistent with the results obtained by RNA-seq (Figure 11D), and a high correlation between the results obtained with these two methods is observed (regression analysis R^2^ = 0.86686), indicating that the RNA-seq results are reliable.

The results indicated that *cis*-abienol induction could enhance the MAPK cascade in tomato roots, promote JA and SA signal transduction, leading to the upregulation of phytoalexin synthesis-related genes, and the tomato resistance to bacterial wilt was ultimately enhanced.

## 3. Discussion

### 3.1. Extracellular Signal Conversion Induced by cis-Abienol

The activities of antioxidant enzymes such as CAT, POD, and SOD are closely related to the content of ROS, which plays an important role in plant disease resistance [27]. Specifically, ROS can activate sensors at the apoplast and induce Ca^2+^ flow from outside the cell membrane into the membrane. Signals received on the cell surface are transmitted into the cell via spatiotemporal changes in Ca^2+^ concentrations in the cytoplasm, and Ca^2+^ signals and ROS signalling pathways could be mutually reinforced. Wang et al. (2022) [28] and Zheng and Li (2022) [29] found that immune elicitors such as BTH and SO_2_ could increase the activity of tomato antioxidant-related enzymes and increase ROS levels. ROS act as defence-signalling substances that upregulate the expression of disease resistance-related genes in plants. We found that the expression of the calcium-binding protein gene (Solyc01g058720.3) was upregulated after *cis*-abienol treatment, and the activities of CAT, POD, and SOD increased. These results suggest that the *cis*-abienol treatment may trigger intracellular defence response by regulating in vivo ROS levels and changing the intracellular Ca^2+^ concentration in tomato roots.

### 3.2. Intracellular Signal Transduction Induced by cis-Abienol

MAPK cascade signalling plays an important role in plant-induced resistance, eliciting signals induced by changes in ROS and Ca^2+^ content to facilitate intracellular transmissions. MAPK is activated and phosphorylates different effector proteins to enter the nucleus to act on transcription factors and regulate gene expression [30]. *SlMPK2* and *SlMPK3* are key genes in MAPK signalling, and silencing these genes negatively impacts MAPK signalling, decreasing the resistance to bacterial wilt [31]. Sclareol, which has a similar chemical structure as *cis*-abienol, exerts its resistance by affecting *SlMPK3* expression in *Arabidopsis thaliana* [16]. In this study, the expression of *SlMPK2* and *SlMPK3* genes was upregulated in tomato roots induced by *cis*-abienol, indicating that *cis*-abienol can induce the MAPK cascade in tomato cells, transmit the signal to the nucleus and induce the expression of defence-related genes.

### 3.3. Signal Transmission between Cells Induced by cis-Abienol

The MAPK cascade induces the expression of defence genes, which in turn trigger the SA and JA signal transduction pathways. SAR mediated by SA and ISR mediated by JA differ from each other in the regulation of pathways, production of immune elicitors, and induction of defence genes. For example, the defence response induced in recipient plants by isoprene is dependent on SA signalling; in contrast, sesquiterpene-caryophyllene induces resistance through JA signalling [32]. Hesperidin-induced resistance to tobacco bacterial wilt depends on the SA pathway [33], whereas sclareol induces resistance to bacterial wilt by affecting the JA pathway in *Arabidopsis thaliana* [16]. However, the defence pathways of SA- and JA-dependent compounds have been found that can interact with each other and synergistically increase. For example, silicon-induced resistance to bacterial wilt in tomatoes may involve the SA and JA pathways [10], and tryptophan can trigger the production of SAR and ISR in potatoes [34]. In this study, the expression of key genes *SlJAZ2* and *SlJAZ3* in JA synthesis were upregulated by *cis*-abienol, and the contents of JA and SA in tomato roots increased, but the extent of increase and duration of this increase in JA content were greater than those of SA. Seo et al. (2012) [16] reported that *cis*-abienol only depended on the JA signalling pathway, but our study reveals that *cis*-abienol-induced resistance not only mainly relies on the JA signalling pathway, but also the SA signalling pathway is also involved, which is consistent with the results of Zhao and colleagues (2022) [34]. Seo et al. (2012) [16] may not have conducted a comprehensive analysis of relevant metabolic pathways, only at the genetic level. To confirm our conclusion, we will continue to combine proteomic and metabolomic analysis of the molecular mechanism.

### 3.4. Production of Phytoalexins Induced by cis-Abienol

The signal transduction of SA and JA is closely related to the phenylpropane metabolic pathway. PAL and PPO are key enzymes of the phenylpropane metabolic pathway in plants, which are involved in the synthesis of resistant substances, such as SA, lignin, and flavonoids. POD is involved in the final step of lignin biosynthesis. MacIntyre et al. (2022) [11] showed that seaweed polysaccharides could induce an increase in PAL and POD activities in tomato seedlings, resulting in an increase in lignin content and the enhancement of bacterial wilt resistance. In this study, we found that the expression of *SlPAL*, *Sl4CL*, and *SlCHI1* in tomato roots was upregulated after day 1 of *cis*-abienol treatment, and the activities of PAL, POD, and PPO increased within 1–6 days after treatment. Additionally, we showed that the contents of phytoalexin flavonoids and lignin in tomatoes were high 3–6 days after treatment. Moreover, results of the induction time interval (Figure 4) also revealed that the optimal resistance of tomato to bacterial wilt was achieved after 3–6 days of *cis*-abienol treatment.

## 4. Materials and Methods

### 4.1. Collection of Seeds and Pathogens

Tomato seeds (*S. lycopersicum* cv. 72-69) were provided by the Institute of Vegetables Qingdao Academy of Agricultural Sciences. When the plants grew three leaves, they were transplanted into a pot (120 mm in diameter × 165 mm in height) filled with nutrient soil, grown in a greenhouse with natural light at the Qingdao experimental base of the Tobacco Research Institute of the Chinese Academy of Agricultural Sciences (Jimo, China).

*R. solanacearum* Y-45 (−80 °C refrigerator preservation) plates were purchased from the Agricultural Culture Collection of China. Bacterial colonies were activated in Nutrient Broth Medium (Solarbio Co., Beijing, China). After activation, a total of 100 μL of *R. solanacearum* suspension was cultured in a Nutrient Agar (NA) medium (Solarbio Co.) at 28 °C. After 36 h of culture, the single colonies of *R. solanacearum* were isolated from the NA medium, placed in the shaker (28 °C, 200 rpm), and cultured until OD_600_ = 0.1 (approximately 1 × 10^8^ CFU/mL).

### 4.2. Isolation and Identification of cis-Abienol

*cis*-Abienol was extracted from the inflorescence of tobacco (*N. tabacum* cv. Samsun NN) and purified in our laboratory. The purity of the product, as assessed by high-performance liquid chromatography (HPLC), was >95%. Tobacco was grown in the southwest experimental base of the Tobacco Research Institute of the Chinese Academy of Agricultural Sciences (Xichang, China). Inflorescences were collected during the full bloom period, and glandular trichome secretions were obtained according to the method of Yang and colleagues (2022) [13]. The extract was dissolved in 95% ethanol and filtered through a 0.22 μm filter membrane for preparation. The following conditions were followed for the HPLC procedure: column SUNFIRE PREP C18 OBDTM (19 mm × 250 mm, 5 μm); UV detector detection wavelength 237 nm; column temperature 35 °C; flow velocity 10 mL/min. Mobile phase: the target components were collected after initial purification with 90% MeOH-H_2_O (Macklin Biochemical Co., Ltd., Shanghai, China, purity: LC), followed by secondary purification with 90% MeCN-H_2_O (Macklin Biochemical Co., Ltd., purity: LC). The solvent was removed under reduced pressure (45 °C) and freeze-dried (−70 °C) to obtain the white-powdered *cis*-abienol. The separated *cis*-abienol was identified by the UPLC, as described by Fu and colleagues (2020) [35] (Figure S1).

Because *cis*-abienol is insoluble in water, a microemulsion was formulated for agricultural use; the conditions were as follows: 1% *cis*-abienol was weighed, followed by the addition of 20% ethanol and 7% n-butanol, stirred evenly, 20% emulsifier (AEO-9: SDS, 3:1) was added, stirred evenly to make it transparent, and 52% H_2_O was added to replenish.

### 4.3. In Vitro Activity against R. solanacearum of cis-Abienol

The growth inhibition rate of *cis*-abienol against *R. solanacearum* was determined using the Oxford cup method. *cis*-Abienol (100 mg) was accurately weighed and prepared in 10 mg/mL mother liquor with 95% ethanol. The original liquor was diluted with 95% ethanol and prepared into 10, 20, 40, 60, 80, and 120 μg/mL solutions of *cis*-abienol. The 300 mL NA medium was sterilised by autoclaving (121 °C, 25 min) and cooled to approximately 45–50 °C. Subsequently, 10 mL of *R. solanacearum* solution was added (OD_600_ = 1.0) and mixed thoroughly. Finally, the uncoagulated culture medium was poured into the 9 cm diameter Petri dishes evenly, and each dish contained approximately 15 mL. In the middle of the dish, the Oxford cup was placed vertically and pressed lightly, with no gap between the cup and the surface of the medium. A total of 100 μL of *cis*-abienol solution was poured into the Oxford cup, and 95% ethanol served as the mock solution. Seven treatments were performed, and each treatment was repeated four times. The culture mediums were placed in an incubator at 28 °C for 48 h. The presence or absence of a bacteriostatic ring was observed, and its diameter was determined by the cross-over method.

### 4.4. Determination of the Suitable Conditions of Resistance to Bacterial Wilt Induced by cis-Abienol

#### 4.4.1. Determination of the Suitable Organ for Induction

Treatment with *cis*-abienol at 20 μg/mL and 60 μg/mL was carried out by root drenching and foliar spraying. Water treatment was used as the negative control. A total of six treatments were performed; each treatment was repeated four times, with each repetition of 12 tomato seedlings, in a random block arrangement. Tomato seedlings at the third-leaf stage were transplanted into the pots (120 mm in diameter × 165 mm in height) containing 0.8 kg of sterilised peat soil. At the sixth-leaf stage, the foliar spraying group was sprayed with *cis*-abienol solution until the drops dripped, while the root drenching group was poured with 20 mL *cis*-abienol solution per plant. After 24 h of application, 10 mL of the activated inoculation solution of *R. solanacearum* was inoculated onto the roots of tomato seedlings. After inoculation, tomato seedlings were grown in an artificial climate chamber at a temperature of 28 °C, 14 h light/10 h darkness, and 70% relative humidity. The infection of tomato seedlings was inspected 7 days later. The disease index and relative control efficiency were calculated according to the procedure described Table 3.

Disease index = [∑ (number of plants in each disease grade × disease grade)/(number of plants in the highest disease grade × total number of plants)] × 100.

Relative control efficiency (%) = (control disease index − treatment disease index)/control disease index × 100.

#### 4.4.2. Determination of the Optimal Concentrations for Resistance Induction

The effect of 10, 20, 40, and 60 μg/mL *cis*-abienol was assessed; zhongshengmycin (Fujian Kaili Biological Products Co., Ltd., Zhangzhou, China) at 50 μg/mL was used as a positive control and water served as the negative control, with a total of six treatments. Each treatment was repeated four times, with each repetition of 12 tomato seedlings, in a random block arrangement. The procedure for applying the *cis*-abienol, inoculating bacteria, greenhouse environment, and disease investigation were the same as those in the root-drenching treatment group.

#### 4.4.3. Determination of Optimal Time Intervals for Resistance Induction

The concentration of *cis*-abienol used was 60 μg/mL. After *cis*-abienol was applied, four time intervals of inoculation were designed: 1, 3, 6, and 10 days. Water served as the negative control in all treatment intervals, and a total of eight treatments were prepared; each treatment was repeated four times, with each repetition of 12 tomato seedlings, in a random block arrangement. The procedure for applying *cis*-abienol, inoculating bacteria, greenhouse environment, and disease investigation was the same as those for the root-drenching treatment group.

#### 4.4.4. Determination of Optimal Times for Resistance Induction

A concentration of 60 μg/mL of *cis*-abienol was used for this study. The treatment was carried out one, two, and three times, with a 3-day interval between each induction; water served as the negative control. A total of four treatments were prepared, and each treatment was repeated four times with 12 tomato seedlings in a random block arrangement. The application of *cis*-abienol, bacterial inoculation, greenhouse conditions, and disease investigation was identical to those of the root-drenching treatment group.

### 4.5. Determination of Development Period and Agronomic Characters of Tomato Seedlings Induced by cis-Abienol

Two concentrations of *cis*-abienol (20 μg/mL and 60 μg/mL) were applied to irrigate the roots, and the roots were irrigated with water as the negative control. There were three treatments, and each treatment was repeated four times, with each repetition of 12 tomato seedlings, in a random block arrangement. Tomatoes with two or three leaves were transplanted. *cis*-Abienol was applied on days 10 and 25 after transplantation, with 20 mL for both times and was placed in a natural greenhouse. Agronomic traits were noted on the 70th day after transplanting. Five plants were selected from each replicate; the plant height was measured using a tape measure, and the fresh weight of the above-ground and underground parts of the plants was determined using an electronic balance after the plants were washed.

### 4.6. Determination of Main Defence Enzyme Activities and Phytoalexin and Phytohormone Content in Tomato Roots

#### 4.6.1. Experimental Design

The experiment was carried out when the tomato seedlings were at the sixth-leaf stage after transplanting. The concentrations of *cis*-abienol microemulsion were 20 and 60 μg/mL, and water was used as the control; a total of three treatments were performed, each treatment was repeated three times, and each treatment was repeated with 60 tomato seedlings, randomly arranged in groups. The application mode was the same as that of the root-drenching treatment group. On the 1st, 3rd, 6th, 10th, and 15th day after the application of *cis*-abienol, four tomato seedlings with the same growth were selected in each treatment, and their roots were separately washed with distilled water, stored in liquid nitrogen, and used for measurements.

#### 4.6.2. Determination Methods

The main defence enzymes included POD, catalase CAT, PPO, SOD, and PAL, and the activities of defence enzymes were determined using the kit method (Shanghai Shenggong Bioengineering Co., Ltd., Shanghai, China). Major phytoalexins, including total flavonoids and lignin, were determined using sodium nitrite-aluminium nitrate colourimetry following the methods of Pastrana-Bonilla and colleagues (2003) [36]. The lignin content was determined using the acetyl bromide assay based on Zhong and colleagues (2015) [37]. Major plant hormones, including SA and JA, and SA and JA contents were determined using an ELISA kit (Shanghai Abimat Pharmaceutical Technology Co., Ltd., Shanghai, China).

The chemical reagents for the above tests were purchased from Sinopharm Chemical Reagent Co., Ltd., Shanghai, China, purity: GR.

### 4.7. Transcriptome Sequencing of Tomato Roots

#### 4.7.1. Experimental Design

Tomato seedlings were hydroponically treated as described by Debouba and colleagues (2007) [38]. At the sixth-leaf stage, the treatment was carried out with *cis*-abienol microemulsion 60 mg/L (T) and water as negative control (CK). Each treatment was repeated three times, with six seedlings per repetition, and a random block arrangement was carried out. After 24 h of application, the roots of the tomato seedlings per repeat were washed with distilled water and immediately stored in liquid nitrogen.

#### 4.7.2. RNA Extraction and Transcriptome Sequencing

A QIAGEN polysaccharide polyphenol kit was used to extract RNA from treated and control group samples, and an Agilent 2100 bioanalyzer was used for quality control. Paired-end libraries were constructed and sequenced using Illumina NovaSeq 6000 (referred to FA download link: ftp://ftp.ensemblgenomes.org/pub/plants/release-54/fasta/solanum_lycopersicum/dna/ (accessed on 5 June 2022); GTF download link: ftp://ftp.ensemblgenomes.org/pub/plants/release-54/gtf/solanum_lycopersicum/ (accessed on 5 June 2022)) in the Ensymbl Database. DESeq2 software (1.20.2) identified DEGs (|log_2_ (FoldChange)| ≥ 1) between T and CK of tomato seedlings, clusterProfiler software performed GO, and Kyoto Encyclopedia of KEGG pathway enrichment analysed differential genes. The related work of RNA extraction and transcriptomic sequencing was undertaken by Nuohe Zhiyuan Technology Co., LTD (Beijing, China). The data have been deposited in the Sequence Read Archive database under the accession number PRJNA994673.

#### 4.7.3. Analysis of Major Differential Gene Validation

Ten plant disease resistance DEGs were screened according to the enrichment pathway analysis of the transcriptome DEGs. Actin was used as an internal reference gene, and its expression level was verified using qRT-PCR. Primer5.0 software was used to design specific primers for the target gene and internal reference gene, and the primers were synthesised by Shanghai Shengong Biology Co., Ltd., Shanghai, China (Table S5). The amplification system of q-PCR was 20 μL and was performed on a QuantStudio 5 fluorescence quantitative PCR instrument (Thermo Fisher Scientific, Waltham, MA, USA). Thermal cycling conditions were as follows: 95 °C for 3 min, followed by 40 cycles, 95° C for 15 s, 60 °C for 1 min, and fluorescence signals were collected at the end of 72 °C. The procedure of the dissolution curve was as follows: denaturation at 95 °C for 15 s, annealing at 55–60 °C for 1 min, denaturation at 95 °C, and collection of fluorescence signal during the process of annealing to denaturation. Relative gene expression levels were calculated using the 2^−ΔΔCt^ method [39], and the β-actin gene was used as the internal reference gene.

### 4.8. Statistical Analysis

Statistical data were analysed using SPSS 19.0 software (IBM, Armonk, NY, USA). Analysis of variance was performed to determine the statistical significance of the difference between indicated values. *p* < 0.05 indicated a significant difference. Duncan’s multiple range test was performed.

## 5. Conclusions

The findings of this research suggest that tobacco *cis*-abienol can be used as an effective immune elicitor for controlling tomato bacterial wilt. The optimal method for inducing tomato resistance to bacterial wilt is root drenching at the concentration of 60 μg/mL, and 2–3 consecutive applications with 3–6 interval days are sufficient to induce effective resistance. *cis*-Abienol-induced bacterial wilt resistance mainly depends on the ISR mediated by the JA signalling pathway, and the SAR mediated by the SA signalling pathway is involved as well. *cis*-Abienol treatment also increases the content of phytoalexins, such as flavonoids and lignin, in tomato roots, which can enhance tomato resistance to bacterial wilt.

This study established a new eco-friendly method for controlling tomato bacterial wilt and highlighted the potential of tobacco *cis*-abienol as a botanical pesticide. Additionally, it provided a basis for alternative utilisation of tobacco plants.

## Figures and Tables

**Table 1:** Effect of *cis*-abienol treatment on the growth and development of tomato seedlings.

*cis*-Abienol (μg/mL)	Sowing to Initial Time of Flowering (d)	Plant Height (cm)	Aboveground Fresh Weight (g)	Underground Fresh Weight (g)
60	47–49	97.25 ± 7.17 a	135.68 ± 10.18 a	30.26 ± 3.10 a
20	49–51	101.37 ± 4.57 a	130.42 ± 9.53 a	29.43 ± 2.49 a
Water	50–51	94.82 ± 6.62 a	127.47 ± 9.42 a	27.46 ± 3.64 a

Different lowercase letters denote statistical significance as determined via Duncan’s multiple range test (*p* < 0.05).

**Table 2:** Tomato root DEGs related to disease resistance after treatment with *cis*-abienol.

Gene ID	Name	Description	Log_2_FC
MAPK cascades			
Solyc08g014425.1	*MPK2*	Mitogen-activated protein kinase 2	1.11
Solyc06g005170.3	*MPK3*	Mitogen-activated protein kinase 3	1.03
Solyc11g005720.1		Protein MKS1	1.91
Solyc08g005050.3	*MYC1*	Transcription factor MYC1	1.35
Solyc02g082920.3	*CHI3*	Acidic 26 kDa endochitinase precursor	3.25
Solyc10g055810.2	*CHI9*	Basic 30 kDa endochitinase precursor	1.92
Solyc01g058720.3		Calcium-binding protein CP1	2.30
Solyc03g096670.3	*PP2C-2*	Protein phosphatase 2C AHG3 homolog	2.58
Plant hormone metabolism			
Solyc12g007230.2	*IAA8*	Auxin-regulated IAA8	1.25
Solyc10g055260.2		LAX5 protein	2.96
Solyc12g009220.2	*JAZ2*	Jasmonate ZIM-domain protein 2	3.35
Solyc03g122190.3	*JAZ3*	Jasmonate ZIM-domain protein 3	4.56
Solyc07g043580.3		Transcription factor PIF4	3.86
Solyc01g102300.3		Transcription factor PIF3 isoform X1	2.07
Solyc05g052980.3		Protein phosphatase 2C 37	1.23
Solyc06g053710.3	*ETR4*	Ethylene receptor ETR4 precursor	3.45
Solyc09g089930.2	*EREB*	Ethylene-responsive element binding protein	3.70
Solyc03g093130.3		Xyloglucan endotransglucosylase-hydrolase XTH3 precursor	−1.69
Solyc00g174330.3	*P4*	Pathogenesis-related leaf protein 4 precursor	5.85
Solyc05g050280.3		Jasmonic acid-amido synthetase JAR1	1.60
Solyc11g011260.1	*GAI*	DELLA protein GAI	−1.20
Solyc00g174330.3	*P4*	Pathogenesis-related leaf protein 4 precursor	5.85
Solyc01g106630.2		PR1 protein precursor	1.52
Phenylpropanoid biosynthesis			
Solyc09g007910.3		Phenylalanine ammonia-lyase	−1.83
Solyc10g086180.2		Phenylalanine ammonia-lyase	3.13
Solyc03g036470.2		Low-quality protein: phenylalanine ammonia-lyase-like	3.13
Solyc05g056170.3	*PAL*	Phenylalanine ammonia-lyase	3.65
Solyc12g042460.2	*4CL*	4-coumarate-CoA ligase	1.59
Solyc05g010320.3	*CHI1*	Chalcone-flavonone isomerase	2.27
Solyc12g055820.2		Probable cinnamyl alcohol dehydrogenase 1	1.32
Solyc10g078540.2		Glutamate dehydrogenase	1.85
Solyc01g098610.3	*GSH2*	Glutathione synthetase, chloroplastic	1.47
Solyc01g080280.3		Glutamine synthetase	−4.92
Solyc03g098240.3		Glutamate decarboxylase	3.01
Solyc01g080280.3	*GS2*	Glutamine synthetase	−4.92
Solyc01g098610.3		Glutathione synthetase, chloroplastic	1.46
Secondary metabolism			
Solyc05g047530.3		Cytochrome P450 CYP73A100	4.54
Solyc01g105590.2	*AT3*	AT3 protein	−7.47
Solyc10g078220.2		Cytochrome P450 98A3	4.70
Solyc01g008110.3	*CYP51*	Sterol C14-demetylase	1.06
Solyc10g078230.2		Cytochrome P450 98A2-like	3.02
Solyc02g093270.3		Caffeoyl-CoA O-methyltransferase-like	2.51
Solyc08g074620.2		Polyphenol oxidase E, chloroplastic	9.80
Solyc07g032740.3		Aspartate aminotransferase, cytoplasmic	3.08
Solyc01g110290.3	*SQS1*	Squalene synthase	1.63
Solyc01g101210.3	*TPS33*	Viridiflorene synthase	2.48
Solyc12g006510.2	*SlTTS1*	Beta-amyrin synthase	4.45

**Table 3:** Disease grade standards.

Disease Grade	Standard
0	No visible disease in any plant part.
1	Chlorotic spots were occasionally observed on the stem or the leaves below 1/2 of the diseased side wilted.
3	Black stripes were observed on the stem but at a height shorter than 1/2 of the stem height or 1/2 to 2/3 of the leaves on the diseased side wilted.
5	Black stripes on the stem exceed 1/2 of the stem height but do not reach the top of the stem, or more than 2/3 of the leaves on the diseased side wilted.
7	Black stripes on the stem reach the top of the stem, or all leaves of the infected plant were completely wilted.
9	The plant almost died.

## Data Availability

The data have been deposited in the NCBI database under the accession number PRJNA994673.

## Supplementary Materials

The following supporting information can be downloaded at: https://www.mdpi.com/article/10.3390/ijms241512226/s1.

## Abbreviations

The following abbreviations are used in this manuscript:

PPO	Polyphenol Oxidase
POD	Peroxidase
PAL	Phenylalanine Ammonia-Lyase
CAT	Catalase
SOD	Superoxide Dismutase
SA	Salicylic Acid
JA	Jasmonic Acid
MAPK	Mitogen-Activated Protein Kinase
BTH	Benzothiadiazole
ROS	Reactive Oxygen Species
ISR	Induced Systemic Resistance
SAR	Systemic Acquired Resistance
GO	Gene Ontology
KEGG	Genes and Genomes
NA	Nutrient Agar
HPLC	High-Performance Liquid Chromatography
