# Supplementary material for: Roles and Preliminary Mechanism of Tobacco cis-Abienol in Inducing Tomato Resistance against Bacterial Wilt
Source: Int J Mol Sci. 2023 Jul 31;24(15):12226. doi: 10.3390/ijms241512226 (PMC10418768; doi:10.3390/ijms241512226)
Supplement: Supplementary file 1 [file ijms-24-12226-s001.zip › Figure S1.pdf]

Tobacco extract

↓

Dissolved in 95% ethanol

↓

Reverse medium pressure preparation apparatus (90% Methanol: 10% H_2O)

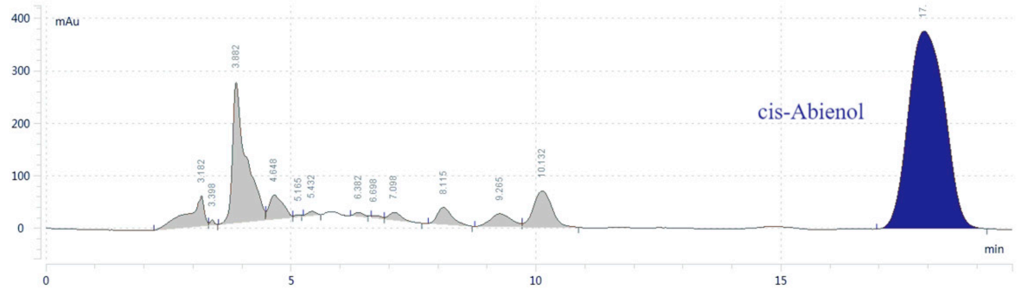

↓

Reverse medium pressure preparation apparatus (90% Acetonitrile: 10% H_2O)

↓

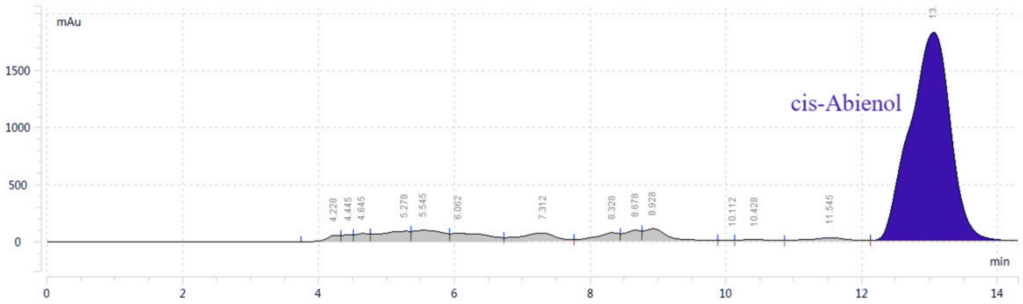

↓

Super High-performance liquid chromatography

↓

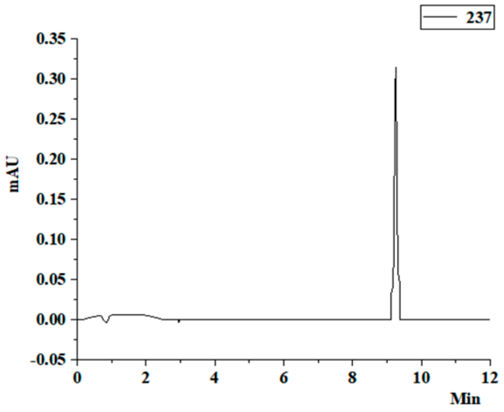

Figure S1. Extraction and purification of cis-abienol.
